# Frequency and molecular epidemiology of class A ESBLs producing Enteroinvasive *Escherichia coli* (EIEC) isolates among patients with diarrhea 

**Published:** 2020

**Authors:** Ahmad Farajzadeh-Sheikh, Mohammad Savari, Hossein Hosseini Nave, Khadijeh Ahmadi Ahmadi, Maryam Afzali

**Affiliations:** 1 *Department of Microbiology, School of Medicine, Ahvaz Jundishapur University of Medical Sciences, Ahvaz, Iran*; 2 * Infectious and Tropical Diseases Research Center, Health Research Institute, Ahvaz Jundishapur University of Medical Sciences, Ahvaz, Iran*; 3 *Department of Microbiology and Virology, School of Medicine, Kerman University of Medical Sciences, Kerman, Iran*

**Keywords:** Enteroinvasive Escherichia coli, Diarrhea, ESBLs, MLVA

## Abstract

**Aim:**

This study aimed to investigate the frequency and molecular epidemiology of class A ESBLs producing Enteroinvasive *Escherichia coli* (EIEC) isolates among patients with diarrhea.

**Background:**

Antibiotic resistance is widespread among diarrheagenic *Escherichia coli* (DEC) in developing countries. Information regarding Extended-Spectrum β-Lactamases (ESBLs) in diarrheagenic pathogens should be considered in clinical management when an optimal treatment is required.

**Methods:**

A total of 581 stool samples were collected from patients with diarrhea in Ahvaz, Iran. PCR was used for the presence of the* ipaH *gene to confirm EIEC strains. The antibiotic resistance pattern of all EIEC isolates was determined by the disk diffusion method. EIEC isolates were screened for class A β-lactamase genes. Genotyping of harboring β-lactamase genes was performed by Multi-Locus VNTR Analysis (MLVA).

**Results:**

Among 13 EIEC isolates, 9 isolates (69.2%) were found ESBL positive by double-disk synergy test (DDST) and PCR. Furthermore, *bla*_CTX-M-15_ and *bla*_CTX-M-1_ genes were detected in 77.8% (n=7) and 44.5% (n=4) of the *bla*_CTX-M-1_ group. On the other hand, the *bla*_TEM-1_ gene was detected in 66.6% (n=6). None of the isolates had *bla*_SHV-1_, *bla*_KPC_, or *bla*_GES_ genes. Six MLVA genotypes were identified.

**Conclusion:**

The current study revealed that the presence of ESBLs genes mediates the resistance of EIEC isolates to the majority of antibiotics in this region. The presence of ESBLs genes in different MLVA types showed that one specific clone was not responsible for spreading the EIEC isolates.

## Introduction

 Severe gastroenteritis results in approximately 800,000 deaths annually by some bacteria. Among the bacterial pathogens, diarrheagenic *Escherichia coli *(DEC) is the most frequent (1). Enteroinvasive Escherichia coli (EIEC) is one of the pathotypes of DEC which causes Shigella-like dysentery in both children and adults (2). EIEC, as with Shigella, can cause invasion and penetration to the epithelial cells of the colon, leading to dysentery, which is an important factor in the mortality of children in developing countries (3). Various genes are involved in the pathogenesis of EIEC strains. One of these genes is an invasive plasmid antigen (*ipaH*) gene which is a key virulence factor for EIEC strains (4). The *ipaH* gene is currently used to confirm EIEC, as the gene is present in multiple copies located on both chromosomes and the plasmid (5). Over the last decades, multidrug resistance (MDR) has increased among *E. coli* clinical isolates. MDR phenotype is obtained by many different mechanisms in clinical isolates. One of the major mechanisms that could lead to the spread of MDR phenotype is the production of extended-spectrum β-lactamase (ESBLs) (6). The spread of ESBLs is a global public health problem. ESBLs can develop resistance to antibiotics such as third and fourth-generation cephalosporins and monobactams (7). The β-lactamases are divided into four categories; A, B, C, and D, based on the Ambler classification. The CTX-M, TEM, SHV, GES, and KPC enzymes belong to class A. These enzymes are encoded by mobile genetic elements usually located on plasmids causing various types of ESBLs to spread globally (8). Among ESBLs, the CTX-M group has been reported globally, and CTX-M-15 is widely distributed around the world. KPC and GES enzymes can hydrolyze the carbapenems used for the treatment of MDR infections (9). The clonal distribution nature of strains harboring the ESBLs genes is a real problem and has been confirmed by many studies (10, 11). Genotyping methods provide useful information for generating genetic relationships among bacterial pathogens for epidemiological research and evolutionary studies. Multilocus variable-number tandem repeat analysis (MLVA) is a PCR-based typing technique used for distinguishing between bacterial isolates. In recent years, various studies have been conducted on Variable Number Tandem Repeat (VNTR) loci variations to discriminate different *E. coli* strains (12). According to previous studies, in comparison with other typing methods such as pulsed-field gel electrophoresis (PFGE) and multilocus sequence typing (MLST), this method is a rapid and low-cost genotyping method which can differentiate closely related strains of bacteria from each other. The MLVA also allowed us to establish associations between genotypes and parameters such as ESBLs genes and multidrug-resistant (4, 12). Although EIEC is an important etiologic agent of diarrhea in different parts of the world, very few reports are available about the occurrence of ESBLs among them and there is no information about the genetic diversity of EIEC strains harboring β-lactamase genes in Iran. Since the clonal circulation of the EIEC strains in our hospital is unclear, thus, this study aimed to investigate the frequency and molecular epidemiology of class A ESBLs producing EIEC isolates among patients with diarrhea. 

## Methods

**Ethical statement**

This study was approved by the Ethics Committee of Ahvaz Jundishapur University of Medical Sciences (IR.AJUMS.REC.1396.434), Ahvaz, Iran.

**Bacterial isolates**

In this cross-sectional study and using the Cochran formula, 581 stool samples were collected from patients with diarrhea referring to the teaching hospitals (Golestan and Abouzar hospitals), in Ahvaz, Iran, from September 2016 to August 2017. Patients with a history of fever, vomiting, nausea, abdominal cramps, watery and bloody diarrhea were included in our study. Also, they had not used the antibiotic nearly 2 weeks before. If patients had taken antibiotics in the last two weeks, they would be excluded from our study.

Stool samples were initially cultured on Hektoen enteric agar and MacConkey agar (Merck, Germany). After overnight incubation at 37°^C^, lactose positive colonies were tested by standard biochemical and bacteriological tests such as Triple Sugar Iron agar (TSI), Christensen’s urea agar, Indole test, Methyl red and Voges-Proskauer tests, and Simmons citrate agar (Merck, Germany) for detection of *E. coli *isolates (3). All isolates confirmed as *E. coli* were preserved in Tryptic Soy Broth (TSB) (Merck, Germany), containing glycerol (30%) at -70°^C^.

**Molecular confirmation of EIEC strains**

All *E. coli* isolates were confirmed as EIEC through amplifying the *ipaH* gene (4). DNA extraction of *E. coli* isolates was performed by the boiling method previously described (11). The sequences of primers used for the detection of the *ipaH* gene are shown in Table 1. Regarding the volume, the reaction was performed in a final volume of 25 μl containing 1X PCR buffer, 1U Taq polymerase, 1 μM MgCl_2_, 200 μM of dNTP (CinnaGen, Iran), 0.2 μl of each primer, and 3 μl of DNA template. The amplification reaction was programmed by a thermal cycler (Eppendorf, Germany) as follows: Initial denaturation at 94 °C for 5 min, 35 cycles of 94 °C for 30 s, annealing 61°C for 45 s, extension 72°C for 1min and final extension 72°C for 5 min. The PCR products were separated on a 1.5% agarose gel containing ethidium bromide and finally visualized in the gel documentation system (Protein simple, USA). *Shigella flexnery* ATCC 12122 was used as a positive PCR control for the *ipaH* gene.

**Antibiotic susceptibility testing**

Antibiotic susceptibility testing was performed on all EIEC isolates tested by the Kirby-Bauer disk diffusion method on Muller-Hinton agar medium (Merck, Germany), based on the Clinical and Laboratory Standard Institute (CLSI) guidelines 2018 (13). The antibiotics included cefotaxime (30 μg), ceftriaxone (30 μg), ceftazidime (30 μg), amikacin (30 μg), imipenem (10 μg), ciprofloxacin (5 μg), colistin sulfate (10 μg), gentamicin (10 μg), aztreonam (30 μg), Sulfamethoxazole-trimethoprim (25 μg), and tetracycline (30 μg) (Mast, UK). *E. coli* ATCC 25922 strain was used as quality control. The phenotype of EIEC was defined as MDR according to the International Expert proposal for Interim Standards Guidelines (14).

**Phenotypic detection of carbapenemase**

Imipenem-resistant strains were screened for carbapenemase production using the modified Hodge test (MHT) (13).

**Phenotypic detection of ESBLs production**

DDST is recommended by CLSI for the phenotypic characterization of ESBL-producing isolates (13). All EIEC isolates were tested by combination disk tests with cefotaxime and ceftazidime (30 μg), with and without clavulanic acid (10 μg), as described by the CLSI guidelines 2018. According to that, a ≥ 5 mm increase in the inhibitory zone diameter for cefotaxime or ceftazidime in combination with clavulanic acid versus the zone diameter of the antimicrobial agent alone is defined as ESBL producer (13). *Klebsiella pneumonia* ATCC 700603 and *E. coli* ATCC 25922 were used as positive and negative controls, respectively. 

**Table 1:** Primers used in this study to detect *ipaH* and ESBLs genes

Gene	Primer Sequence (5´-3´)	Amplicon Size (bp)	Annealing Temperature(°C)	Reference
*ipaH*	F-GAAAACCCTCCTGGTCCATCAGG R-GCCGGTCAGCCACCCTCTGAGAGTAC	437	61	4
*bla*_CTX-M-1_ Group	F-GGTTAAAAAATCACTGCGTC R-TTGGTGACGATTTTAGCCGC	863	54	
*bla*_CTXM-15_	F-CACACGTGGAATTTAGGGACT R-GCCGTCTAAGGCGATAAACA	995	55	
*bla*_CTX-M-1_	F-GGTTAAAAAATCACTGCGTC R-TTGGTGACGATTTTAGCCGC	850	60	
*bla*_TEM-1_	F-GCTATGTGGCGCGGTATTAT R-AAGTTGGCCGCAGTGTTATC	189	56	
*bla*_SHV-1_	F-CCTCATTCAGTTCCGTTTCC R-CCGCGTAGGCATGATAGAAA	389	56	
*bla*_KPC_	F-CGTCTAGTTCTGCTGTCTTG R-CTTGTCATCCTTGTTAGGCG	538	59	
*bla*_GES_	F-ATGCGCTTCATTCACGCAC R-CTATTTGTCCGTGCTCAGG	860	59	

**Table 2:** Locus-specific PCR primers selected for MLVA

Locus	Primer Sequence	Repeat Size (bp)	AnnealingTemperature (°C)
*ms06 *	F-AAACGGGAGAGCCGGTTATT R-TGTTGGTACAACGGCTCCTG	39	57
*ms07*	F-GTCAGTTCGCCCAGACACAG R-CGGTGTCAGCAAATCCAGAG	39	57
*ms09*	F-GTGCCATCGGGCAAAATTAG R-CCGATAAGGGAGCAGGCTAGT	179	57
*ms11*	F-GAAACAGGCCCAGGCTACAC R-CTGGCGCTGGTTATGGGTAT	96	57
*ms21*	F-GCTGATGGCGAAGGAGAAGA R-GGGAGTATGCGGTCAAAAGC	141	57
*ms23*	F-GCTCCGCTGATTGACTCCTT R-CGGTTGCTCGACCACTAACA	375	57
*ms32*	F-TGAGATTGCCGAAGTGTTGC R- AACTGGCGGCGTTTATCAAG	101	57

**Table 3:** Distribution EIEC strains according to seasons, age, gender and clinical symptoms

Strain ID	Sex	Age (month)	Seasonality	clinical symptoms
EIEC 1	Female	0-12	Spring	Watery stool, Abdominal pain, Vomiting
EIEC 2	Female	0-12	Spring	Watery stool, Abdominal pain, Fever
EIEC 3	Male	0-12	Summer	Watery stool, Vomiting, , Fever
EIEC 4	Male	13-24	Summer	Watery stool, Abdominal pain,
EIEC 5	Male	0-12	Summer	Watery stool, Abdominal pain, Fever
EIEC 6	Female	49-60	Summer	Watery stool, Abdominal pain
EIEC 7	Male	0-12	Summer	Abdominal pain, Vomiting
EIEC 8	Female	0-12	Spring	Watery stool
EIEC 9	Male	13-24	Winter	Watery stool, Abdominal pain
EIEC 10	Male	0-12	Summer	Watery stool, Abdominal pain, Vomiting
EIEC 11	Female	49-60	Summer	Watery stool
EIEC 12 EIEC 13	Male Male	13-24 0-12	Winter Spring	Abdominal pain, Vomiting Watery stool, Fever

**Molecular Detection of β-Lactamase Genes**

PCR was performed for detection of *bla*_CTX-M-1, _*bla*_CTXM-15, _*bla*_TEM-1_*, bla*_SHV-1_*, bla*_KPC, _and *bla*_GES _genes. The PCR conditions were similar to previous studies (11, 15-17). The specific primers and annealing temperatures of ESBLs genes are listed in Table 1.

**Genotyping of ESBLs producing EIEC isolates by MLVA analysis**

MLVA was performed for ESBLs genes-harboring EIEC isolates. Seven VNTR loci were selected for the genetic typing of the EIEC isolates. PCR was carried out as described by a previous study (18). The primers and repeat sizes for each locus are shown in Table 2. The PCR products were electrophoresed on a 1.5% agarose gel containing ethidium bromide and visualized in a gel documentation system (Protein simple, USA). The copy numbers of the repeat for each isolate were calculated by the following formula:

Number of repeats = size of each locus (bp) – flanking regions (bp)/repeat size (bp).

The results were imported into Microsoft Excel 2010 software and analyzed by the Bionumerics Software v.6.6 (Applied maths, Sint-Martens-Latem, Belgium). For clustering, a cut-off value of 90% similarity was used. The dendrogram of genetic relationships was generated using the Unweighted Pair Group Method with Average linkages (UPGMA) (19).

**Statistical analysis**

The results were analyzed, using SPSS version 16 to obtain frequencies and comparisons among clones. A nonparametric chi-square test was also applied. A P.value of < 0.05 was considered statistically significant. 

## Results

**Study population**

In this descriptive cross-sectional study, 581 stool samples were collected from patients with diarrhea from two teaching hospitals including Golestan and Abouzar hospitals in Ahvaz, Iran. The patients’ age ranged between 0 and 81 years. Out of 581 diarrhea samples, 43.2% (n=251) were DEC strains confirmed by culture and biochemical tests. 

**Distribution of E. coli and EIEC**

Of the 251 DEC, 57.4% (n=144) were isolated from the children and 42.6% (n=107) from adults. The occurrence of DEC strains in males and females was 54.6% (n=137) and 45.4% (n=114), respectively. Of the 144 children, 54.9% (n=79) were male and 45.1% (n=65) were female, and among the 107 adults, 54.2% (n=58) were male and 45.8% (n=49) were female. Out of 251 *E.coli* isolates, 5.1% (n=13) were positive for* ipaH*. All the EIEC strains were retrieved from children. Of 13 strains of EIEC, 84.6% (n=11) isolated from children under the age of 2 years (P<0.05). The distribution of EIEC strains according to age, gender, and clinical symptoms is shown in Table 3.

**Antimicrobial susceptibility test of EIEC strains**

Among 13 EIEC isolates, all isolates were susceptible to ciprofloxacin. The highest rate of antibiotics resistance was found against ampicillin 100% (n=13), followed by trimethoprim/sulfamethoxazole 84.6% (n=11), tetracycline 76.9% (n=10) (Table 4). The MDR phenotype was observed in 76.9% (n=10) of the EIEC isolates. MHT results for two imipenem-resistant isolates showed that both strains were positive for carbapenemase phenotype. The results of antimicrobial susceptibility testing of the 13 EIEC isolates to 12 antibiotics are summarized in Table 4. Our data revealed nine phenotypic patterns of resistance among EIEC isolates (Table 5).

**Phenotypic ESBLs detection**

 Out of 13 EIEC, 69.2% (n=9) isolates were ESBLs producers when tested by the DDST. All EIEC isolates that were positive for ESBLs revealed the MDR phenotypes.

**Detection of β-lactamase genes**

PCR was performed for all EIEC strains. Of 9 ESBL producers, 77.8% (n=7) strains carried the *bla*_CTX-M-1_ group. The *bla*_CTX-M-15_ subtype was identified in all of the *bla*_CTX-M-1_ groups while the *bla*_CTX-M-1_ variant was detected in 44.5% (n=4) isolates of the *bla*_CTX-M-1_ group. On the other hand, the *bla*_TEM-1_ gene was detected in 66.6% (n=6). None of the isolates had *bla*_SHV-1_, *bla*_KPC_, and *bla*_GES_ genes. The *bla*_TEM-1_ and *bla*_CTX-M-1_ genes were found to coexist with *bla*_CTX-M-15_ in 4 isolates.

**MLVA assay**

**Figure 1:**
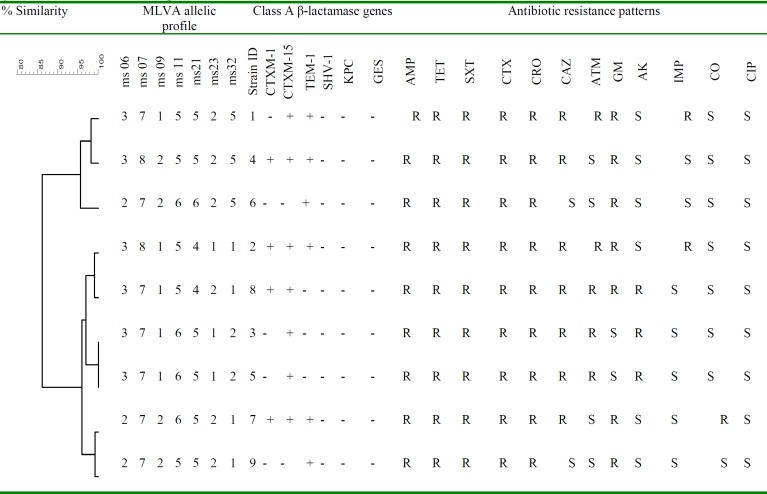
Dendrogram representing the genetic diversity of nine ESBL producer-EIEC Isolates by MLVA; class A β-lactamase genes; Antibiotic resistance patterns

**Figure 2:**
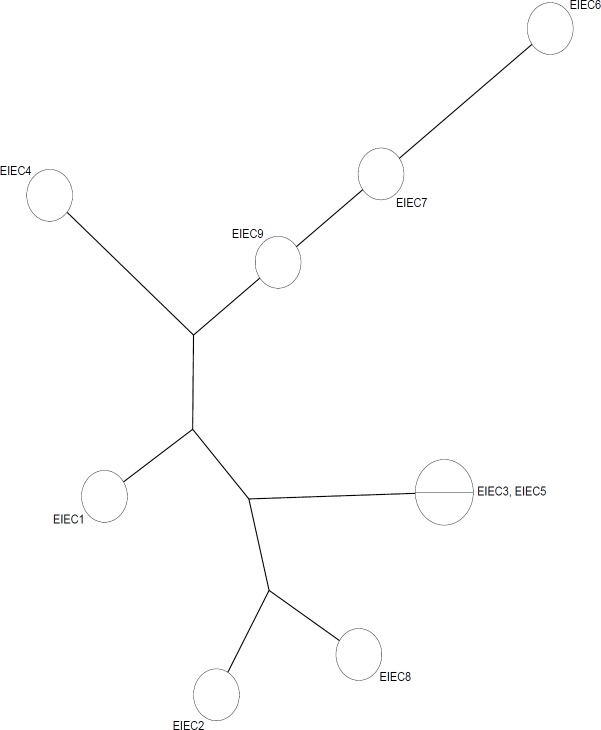
Minimal Spanning Tree of 9 ESBL producer-EIEC Isolates. Categorical coefficient was used to construct the MST, showing the distances and the genetic relationships between the ESBLs genes-harboring EIEC isolates

Analysis of the MLVA profiles using UPGMA showed that nine EIEC ESBL producers isolates were grouped into six distinct MLVA types with two clusters and three singletons, and three multitone genotypes (Figure1). All MLVA- Loci were present in all studied isolates. The minimal spanning tree (MST) of virulence genes distribution among different MLVA patterns is shown in Figure 2. Each circle denotes an MLVA type, with the number of isolates in each type, as indicated within the circle.

**Table 4:** The antibiotic susceptibility testing results of EIEC isolates

Antimicrobial	Susceptible	Resistant
Cefotaxime	4 (30.7%)	9 (69%)
Ceftriaxone	4 (30.7%)	9 (69%)
Ceftazidime	6 (46.1%)	7 (53.8%)
Imipenem	11 (84.6%)	2 (15.3%)
Ciprofloxacin	13 (100%)	0 (0%)
Tetracycline	3 (23%)	10 (76.9%)
Trimethoprim/Sulfamethoxazole	2 (15.3%)	11 (84.6%)
Gentamicin	6 (46.1%)	7 (53.8%)
Amikacin	10 (76.9%)	3 (23%)
Ampicillin	0 (0%)	13 (100%)
Aztreonam	8 (61.5%)	5 (38.4%)
Colistin sulfate	12 (92.3%)	1 (7.6%)

**Table 5:** Antibiotic resistance phenotypic patterns of EIEC isolates

Resistance Pattern	Phenotypic resistance	Number of resistant EIEC isolates (%)
I	*AMP*	2 (15.3%)
II	AMP, SXT	1 (7.6%)
III	AMP, SXT, TET	1 (7.6%)
IV	AMP, SXT, TET, CTX, CRO, GM	2 (15.3%)
V	AMP, SXT, TET, CTX, CRO, CAZ, GM	1 (7.6%)
VI	AMP, SXT, TET, CTX, CRO, CAZ, GM, CO	1 (7.6%)
VII	AMP, SXT, TET, CTX, CRO, CAZ, ATM, AK	2 (15.3%)
VIII	AMP, SXT, TET, CTX, CRO, CAZ, ATM, GM, AK	1 (7.6%)
IX	AMP, SXT, TET, CTX, CRO, CAZ, ATM, GM, IMP	2 (15.3%)

CTX: cefotaxime, CRO: ceftriaxone, CAZ: ceftazidime, AK: amikacin , IMP: imipenem, CO:colistin sulfate, GM: gentamicin, ATM: aztreonam, SXT: Sulfamethoxazole-trimethoprim, TET: tetracycline, AMP: ampicillin

## Discussion

EIEC is one of the causes of childhood diarrhea in our country. Unfortunately, there are limited reports on the prevalence of EIEC in Iran. Due to the inability of conventional culture methods to detect pathogenic E. coli from non-pathogenic isolates, EIEC is usually ignored (3). Our results showed that 5.1% (n=13) of the DEC isolates were EIEC.

This is higher than prevalence rates mentioned in similar reports from Northern Iran (0.5%) (20), south of Iran (4.9%) (21), as well as lower than those mentioned in a report from Shiraz (14.3%) (3). It seems that the different distribution of EIEC strains in various studies is due to the difference in geographic and socioeconomic variables, laboratory mistakes in identifying isolates, time, and study conditions. Most studies have been performed in children under the age of 5 (21-23). In our study, there was no age limit for sample collection, but all EIEC isolates were detected in children. Given the importance of this invasive pathogen, further studies should be focused on why EIEC is prevalent in children in this region. As with our study, Natarajan et al. in India, identified EIEC strains only in children (24). We showed that most of the EIEC strains were isolated from children are under age of 2. Previous studies have also indicated that EIEC is more common in children under 2 years (3, 22), which is similar to our study.

According to the WHO guidelines, antibiotics should not be used regularly for the treatment of diarrhea, especially for unknown causes. Antibiotics should be used in bloody and chronic diarrhea to reduce the duration of the disease and the selection of effective antibiotics for the treatment of the patient is necessary (25). However, antimicrobials are widely used to treat diarrhea in Iran. In the present study, the antimicrobial susceptibility pattern showed that ciprofloxacin had the most effect on EIEC isolates. Nevertheless, ciprofloxacin and other quinolones are not used for children because of the risk of damage to immature joints.

The results of this study revealed that the β-lactams, ampicillin, gentamicin, tetracycline, and trimethoprim/ sulfamethoxazole antibiotics had the minimum activity on EIEC strains. Resistance to these antibiotics was more than 50%. These results are consistent with previous reports (22, 23, 26). The extent of EIEC resistance to first-line drugs such as ampicillin, trimethoprim/sulfamethoxazole, and tetracycline in our study may be based on the misuse and overuse of these antibiotics for the treatment of diarrhea. This may result in the spread of antibiotic-resistant bacteria (27). 

Resistance to some broad-spectrum antibiotics such as colistin, ciprofloxacin, imipenem, and amikacin is limited and can be used to treat MDR diarrheal diseases. In this study, the MDR rates of EIEC strains were evaluated. Our results indicated that MDR rates were higher than in a previous study in Ahvaz (23). It seems that misuse and overuse of antibiotics for the treatment of diarrhea is one of the main causes of high levels of MDR in Ahvaz. The spread of ESBLs is a global health problem. ESBL production is one of the main mechanisms that can spread MDR (17). In our study, the prevalence of ESBLs positive in EIEC isolates was (69.2%), which was lower than another study in Ahaz (83%) (23), but higher than a study in Shiraz (50%) (26). In the last decade, the *bla*_CTX-M_ has been the most common ESBLs among MDR organisms around the world. The *bla*_CTX-M_ genes are effective against cefotaxime. Within the CTX-M-1 group, CTX-M-15 is the most widely disseminated genotype (28). There are few reports about the prevalence of *bla*_CTXM-15_ among diarrheagenic *E. coli*. In a study conducted by Memariani et al in Tehran, the *bla*_CTXM-15_ variant was identified in all of the *bla*_CTXM-1_ positive EPEC isolates (11). In our study, most of the strains produced *bla*_CTX-M-15_. The *bla*_CTX-M-15_ is sometimes associated with other genes, such as *bla*_TEM-1_. In a study conducted by Natarajan et al in India, the prevalence rates of *bla*_CTX-M__, _*bla*_TEM_, and *bla*_SHV_ in EIEC isolates were (33.3%), (33.3%), and (0%) respectively (24). Zhou et al. in China reported that (50%) of EIEC strains among children under 5 years old were positive for *bla*_TEM-1_ (29). In our study, the *bla*_CTX-M-15_gene was found to coexist with *bla*_TEM-1_ and *bla*_CTX-M-1_ genes in four isolates. In the present study, resistance to third-generation cephalosporins was most associated with the CTX-M-1 group. 

None of the isolates had *bla*_SHV_. Neither *bla*_KPC_ nor *bla*_GES_ genes were found in this study. However, the results of MHT revealed that all imipenem-resistant isolates were positive for carbapenemase production. In spite of a significant increase in the resistance of carbapenem strains in Iran, our findings showed that probably, other carbapenem genes such as IMI, OXA, and IMP cause resistance to carbapenems.

The genetic diversity of ESBLs producing EIEC isolates has not been studied until now. This is the first report on the evaluation of MLVA for genotyping of class A ESBL genes-harboring EIEC strains. MLVA has been successfully used for genotyping ESBL produces *E. coli* in Denmark (30). In the current study, all ESBLs producing EIEC isolates were discriminated into six different MLVA types with two clusters. The analysis of the UPGMA cluster showed that there is a high genetic diversity in EIEC strains harboring β-lactamase genes. CTX-M-15 gene was the most prevalent β-lactamase among the ESBLs positive EIEC isolates. The presence of the ESBLs genes in unrelated isolates might be because of the horizontal dissemination of mobile genetic elements in the unrelated isolates. This gave us a vision of the current prevalence and the genetic diversity among ESBLs producing strains of EIEC. In our study, EIEC strains with similar MLVA patterns had similar ESBLs gene profiles and similar antibiotic resistance patterns (strains 3 and 5). However, in some cases, EIEC strains with different MLVA types had similar ESBLs gene profiles (strains 4, 2, and 7). The presence of isolates with similar patterns indicates that clonal spread is also involved in the dissemination of ESBL-producing isolates. This study showed that MLVA is an appropriate typing method for epidemiological studies of *E. coli* species due to rapidity, technical simplicity, and the ability to generate numerical data which can be easily shared among medical laboratories. The use of MLVA for typing ESBL *E. coli* remains limited, with some research published and the lack of a shared database for researchers is a disadvantage.

The current study suggests that EIEC may be an important and unrecognized cause of acute diarrhea in children. Thus, use of the molecular diagnostic methods for detecting EIEC and appropriate treatment can be helpful. Also, molecular epidemiologic studies play a significant role in evaluating transmission ways of the pathogen for infection control. Our investigation revealed the widespread prevalence of multidrug-resistant and ESBL-producing EIEC isolates in Ahvaz. These results indicate that it is necessary to continuously monitor the emergence and spread of ESBL-producing isolates for the use of efficient antimicrobials in clinical practice.
